# Evaluating cells metabolic activity of bioinks for bioprinting: the role of cell-laden hydrogels and 3D printing on cell survival

**DOI:** 10.3389/fbioe.2024.1450838

**Published:** 2024-09-26

**Authors:** Elena Laura Mazzoldi, Giulia Gaudenzi, Paola Serena Ginestra, Elisabetta Ceretti, Silvia Clara Giliani

**Affiliations:** ^1^ Angelo Nocivelli Institute of Molecular Medicine, Department of Molecular and Translational Medicine, University of Brescia, Brescia, Italy; ^2^ Department of Mechanical and Industrial Engineering, University of Brescia, Brescia, Italy

**Keywords:** 3D cell printing, hydrogel, alginate, gelatin, cell viability, ATP

## Abstract

**Introduction:**

Tissue engineering has advanced significantly in recent years, owing primarily to additive manufacturing technology and the combination of biomaterials and cells known as 3D cell printing or Bioprinting. Nonetheless, various obstacles remain developing adequate 3D printed structures for biomedical applications, including bioinks optimization to meet biocompatibility and printability standards. Hydrogels are among the most intriguing bioinks because they mimic the natural extracellular matrix found in connective tissues and can create a highly hydrated environment that promotes cell attachment and proliferation; however, their mechanical properties are weak and difficult to control, making it difficult to print a proper 3D structure.

**Methods:**

In this research, hydrogels based on Alginate and Gelatin are tested to evaluate the metabolic activity, going beyond the qualitative evaluation of cell viability. The easy-to-make hydrogel has been chosen due to the osmotic requirements of the cells for their metabolism, and the possibility to combine temperature and chemical crosslinking. Different compositions (%w/v) are tested (8% gel-7% alg, 4% gel-4% alg, 4% gel-2% alg), in order to obtain a 3D structure up to 10.3 ± 1.4 mm.

**Results:**

The goal of this paper is to validate the obtained cell-laden 3D structures in terms of cell metabolic activity up to 7 days, further highlighting the difference between printed and not printed cell-laden hydrogels. To this end, MS5 cells viability is determined by implementing the live/dead staining with the analysis of the cellular metabolic activity through ATP assay, enhancing the evaluation of the actual cells activity over cells number.

**Discussion:**

The results of the two tests are not always comparable, indicating that they are not interchangeable but provide complementary pieces of information.

## Highlights

• Hydrogel based on Alginate and Gelatin: optimization of printing parameter and crosslinking approach.
• Hydrogel viscosity analyses: direct relation with cell viability.
• ATP analysis: implementing metabolic tests to evaluate cell viability.

## 1 Introduction

Bioprinting is a pioneering technology for the fabrication of biomimetic tissue constructs, expected to provide an effective solution to the long-standing shortage of tissues/organs for transplants (Vijayavenkataraman et al., 2019; Vijayavenkataraman et al., 2018). This technique is also used to produce devices loaded with cells in order to study their behaviour in certain scenarios, such as their reaction to drugs (Badylak and Nerem, 2010; Furman, 2013). Bioprinting has gained prominence for its ability to create complex, three-dimensional tissue structures with high precision. This technology uses a layer-by-layer approach to deposit cells, biomaterials, and growth factors, which is crucial for replicating the intricate architecture of human tissues (Huang et al., 2024; Rasouli et al., 2024). Recent studies highlight how this capability is paving the way for the development of highly personalized medical treatments. For instance, bioprinting can generate patient-specific tissues that are tailored to match individual anatomical and physiological requirements, thus reducing the risk of immune rejection and improving treatment outcomes. Bioinks, the materials used in bioprinting, are equally pivotal (Gungor-Ozkerim et al., 2018; Chen et al., 2023). They are designed to support cell viability and promote tissue formation. Recent advances have focused on developing bioinks that mimic the extracellular matrix, providing an environment conducive to cell growth and differentiation. This has led to improvements in the quality and functionality of bioprinted tissues. For example, bioinks now include natural polymers, synthetic materials, and hybrid formulations that cater to different types of cells and tissue requirements. Additionally, recent literature has explored how bioinks can be engineered to release specific growth factors or genetic materials, further enhancing their functionality. This innovation helps guide stem cells to develop into desired tissue types, thereby improving the effectiveness of tissue engineering efforts. The scalability of bioink production is also becoming more feasible, which is essential for translating these technologies from the laboratory to clinical and commercial applications (Lobo et al., 2021).

Two-dimensional models do not reproduce human physiological conditions faithfully; therefore, it is preferable to use 3D tissue models to provide cells with mechanical stimuli, derived from cell-to-cell and cell-to-matrix interaction. Among the available technologies to produce structures for 3D cell cultures, 3D cell printing is one of the most innovative. 3D printing mainly consists of four different techniques: laser-assisted, stereolithography-based, jetting-based, and extrusion printing systems (Jang et al., 2018). Extrusion-based cell printing exploits a syringe filled with a bioink, usually a hydrogel. Encapsulating cellular components in flowable and biocompatible hydrogel also allows to prevent cellular damage from shear forces during the extrusion process (Kim et al., 2016; Cho and Yoo, 2016; Stanton et al., 2015; Pati et al., 2014). The printing head movements in *x*, *y* and *z* allow the deposition of the hydrogel along a 3D structure. Although the resolution of extrusion-based cell printing is lower than laser or inkjet-based cell printing (∼200 μm), this method can produce 3D tissue-mimetic structures easily and very quickly, with limited damage to cell viability or functionality. Moreover, this technique allows the extrusion of a wide range of hydrogel with different viscosities (Park et al., 2016).

When producing a cell-laden printable hydrogel, some important aspects are to be considered: biocompatibility, printability, and mechanical and structural integrity (Jang et al., 2018; Kim et al., 2016; Cho and Yoo, 2016; Stanton et al., 2015; Pati et al., 2014; Park et al., 2016; Tian et al., 2021). Hydrogels with high printability generally have a high viscosity or crosslinking density, which can lead to reduced biological properties. Moreover, hydrogels with good biofunctionality (*i.e.*, excellent cell proliferation, differentiation, and maturation) can lead to less printability potential. The optimal result is a hydrogel capable to keep the printed shape and to promote biological activity (Choi et al., 2021).

Different polymers, both natural and synthetic, have been applied to successfully fabricate supporting frameworks to engineer clinically relevant and mechanically robust connective tissues, including bone and cartilage (Park et al., 2016), while it is more difficult to obtain soft connective tissues. Among natural polymers, Gelatin and Alginate are the most commonly used to produce hydrogels (Choe et al., 2019; Liu et al., 2019; Sonaye et al., 2022). Gelatin is a natural polymer (Cheng et al., 2022) derived from collagen via acidic (type A) or basic (type B) hydrolysis, thus it is relatively easy to obtain in large quantities from animal sources (*e.g.*, bones, tendons, or skin) as compared to pure collagen. This material has a thermo-reversible gelation behaviour in response to the surrounding temperature, which makes it particularly attractive as a hydrogel for bioprinting. Due to this property, cell-laden Gelatin can easily build up the desired 3D structure by regulating temperature and concentration. In addition, Gelatin has cell-responsive properties, such as the arginine-glycine-aspartate (RGD) peptide as a cell binding motif and matrix metalloproteinase (MMP) recognition sequences for degradation. However, Gelatin offers insufficient structural stability due to its low mechanical strength and high temperature sensitivity; this weak mechanical strength needs to be improved by adding other polymers to form composite hydrogels, complicating the entire process.

Alginate, which is refined from brown seaweed, is one of the most preferable natural polymers to produce hydrogels, owing to its low toxicity, low price, and wide applicability. Alginate-based hydrogels have been applied to engineer a variety of tissues, such as bone, cartilage, and adipose tissues. To facilitate tissue formation, it has been widely used as a hydrogel for extrusion bioprinting because it can be instantly polymerized by mixing it with multivalent cations during the printing process (Jang et al., 2018). This material, unlike Gelatin, lacks cell-responsive properties, so cells tend to aggregate in clusters when only Alginate is used as scaffold (Ahmad Raus et al., 2021). Usually, Alginate is blended with other polymers to ensure its biological functionality. In addition, to further improve mechanical properties and structural stability, chemical cross-linking (such as methacrylation and thiolation) is also used (Choi et al., 2021). On the other hand, Alginate biodegrades when in contact with bodily fluids, due to the exchange reaction with monovalent cations: this feature makes it appealing in tissue engineering, because it makes possible to control the degradation rate (Ahmad Raus et al., 2021).

The blend of Alginate and Gelatin to obtain a hydrogel is undoubtedly the most common for 3D cell culture because of it is easy-to-make, economic, fast, non-toxic, biocompatible and biodegradable; furthermore, this kind of hydrogel is a good option for 3D printing at room temperature (Łabowska M. B. et al., 2021), which is useful for experimental tests. In this work, we chose this combination as the target one to evaluate the relation between printed and not printed cell-laden hydrogel and thus finding the real consequences on cell activity of the printing process. Moreover, each hydrogel viscosity is definitely related to the cell viability and the following findings can show this relation trends on a simple and low-cost base.

The first aspect to assess following 3D printing of cell-laden hydrogels is cell viability, since it represents the limiting factor for all the possible applications. Generally, in literature, cell viability is estimated using imaging on live-dead staining, which is mostly qualitative and does not provide any functional information other than cells being dead or alive. In the best scenarios, cell viability is analyzed with Alamar Blue or similar assays which lack in precision and sensitivity (Park et al., 2016; Tian et al., 2021; Choe et al., 2019; Yunping et al., 2024; Zhang et al., 2021; Lozano et al., 2015; Gong et al., 2020; Ouyang et al., 2016; Kirillova et al., 2017). Due to the undeniable difficult steps required for the assays applications on cells that are included in a hydrogel, more reproducible and specific assays are rarely taken into account. For this reason, the use of different methods in parallel, which take in consideration different cell properties (*i.e.*, morphology, metabolic activity, proliferative capacity, etc.), would hopefully provide a more accurate description of 3D structures and of their impact on cell survival and behavior, as described in (Bikmulina et al., 2022). In literature (Liu et al., 2019; Gaudenzi et al., 2024; Mondal et al., 2019; Fayyazbakhsh et al., 2022; Ninan et al., 2014), Alginate and Gelatin hydrogels were often considered but cell viability was estimated only qualitatively and mainly at early timepoints. Moreover, viability is usually assayed on unrealistic high cell densities for connective tissues, which is primarily made up of extracellular matrix, with a limited number of sparse cells.

The present work aims at the analysis of how the actual cell activity, not only in terms of imaging, is affected by the bioprinting process considering the results of biological characterization performed on cell-laden hydrogels with different compositions and viscosities. Another crucial aspect of this work is the comparison between the printed and not printed cell-laden hydrogels and the 2D standard culture control, rarely included in the bioprinting related literature.

In this paper, structures up to 10.3 ± 1.4 mm have been produced, to evaluate an actual 3D structure instead of a few printed layers (10 mm refers to the commercially available scaffolds for tissue engineering), and cell viability has been evaluated by implementing the classical live/dead staining with cell morphology assessment and with the analysis of the cellular metabolic activity through the adenosine triphosphate (ATP) assay, an integrative approach that is lacking in the literature on the topic. The ATP assay shows cell metabolism and proliferation with good reproducibility and sensitivity when cells are grown over several days, making it particularly useful for the measurement of viability with low cell numbers. In fact, in this work, the hydrogels have not been laden with an unmotivated high amount of cells for connective tissues but with an average density of 4 × 10^5^ cells/mL, similar to what has been reported for dermal fibroblasts (1 × 10^5^–5 × 10^5^/ml), for adipocytes in adipose tissue (7 × 10^5^–8–10^5^/ml), and in particular for hyalocytes in the vitreous body of the eye (1 × 10^5^–2 × 10^5^/eye) (Chopra et al., 2016; Eto et al., 2009; Donati et al., 2014). Moreover, ATP differs from imaging analysis, providing a direct information on the amount of cells that are not only alive, but also metabolically active.

A relation between medium viscosity and cell metabolic activity was also highlighted. Even if this hydrogel has been widely used in literature and cell viability is always considered as an initial outcome of bioprinting related research, this novel approach will give a better understanding of cell behaviour in natural-based hydrogels in their most used percentages and combinations and at cell density more similar to the one found in real connective tissues, with an innovative focus on the difference between printed and not printed constructs and with a direct relation of cell viability and 3D structures that strongly influence biological response.

## 2 Materials and methods

### 2.1 Hydrogel composition

Gelatin (Gelatin from porcine skin type A, 300 Bloom, Sigma Aldrich, Saint Louis, MO), Alginate (Alginic sodium salt, 120,000–190,000 g/mol, Sigma Aldrich), distilled water (milliQ), and Iscove’s Modified Dulbecco’s Medium (IMDM powder, Gibco™, Thermo Fisher Scientific, Waltham, MA) were combined to produce the hydrogels. Specifically, milliQ water and Alginate were added and mixed in a graduated cylinder; Gelatin was then added and mixed to the other components. The cylinder has then been autoclaved at 121°C for 30 min, in order to make the mix homogenous and sterile. Finally, 5 mL of IMDM 10X and a proper amount of milliQ water were added when the temperature decreased, to reach the volume of 50 mL. At that point, the hydrogel was ready to be transferred into syringes for the printing tests. The tested compositions (%w/v) were G8A7 (8% Gelatin 7% Alginate), G4A4 (4% Gelatin 4% Alginate), G4A2 (4% Gelatin 2% Alginate). Specifically, G8A7 was printed with a Pressure (P) ranging from 0.7 to 1.8 bars and a printing velocity (V) ranging from 10 to 30 mm/s at a Temperature (T) of 20°C. G4A4 and G4A2 hydrogels were printed with P ranging from 0.8 to 1 bar, V ranging from 20 to 30 mm/s at a T of 10°C. These combinations, especially at higher percentages, have been firstly selected on the basis of the recent literature (Di Giuseppe et al., 2018; Wang et al., 2021; Łabowska MB. et al., 2021) since these hydrogels are demonstrating similar properties (mechanical, chemical, etc.) to native connective tissue, and subsequently to exploit and explore the potential of different combinations to induce differences in the hydrogel viscosity to investigate in relation with cell survival (Cadamuro et al., 2023; Kaliampakou et al., 2023).

### 2.2 Printing, crosslinking and shape optimization

**FIGURE 1:**
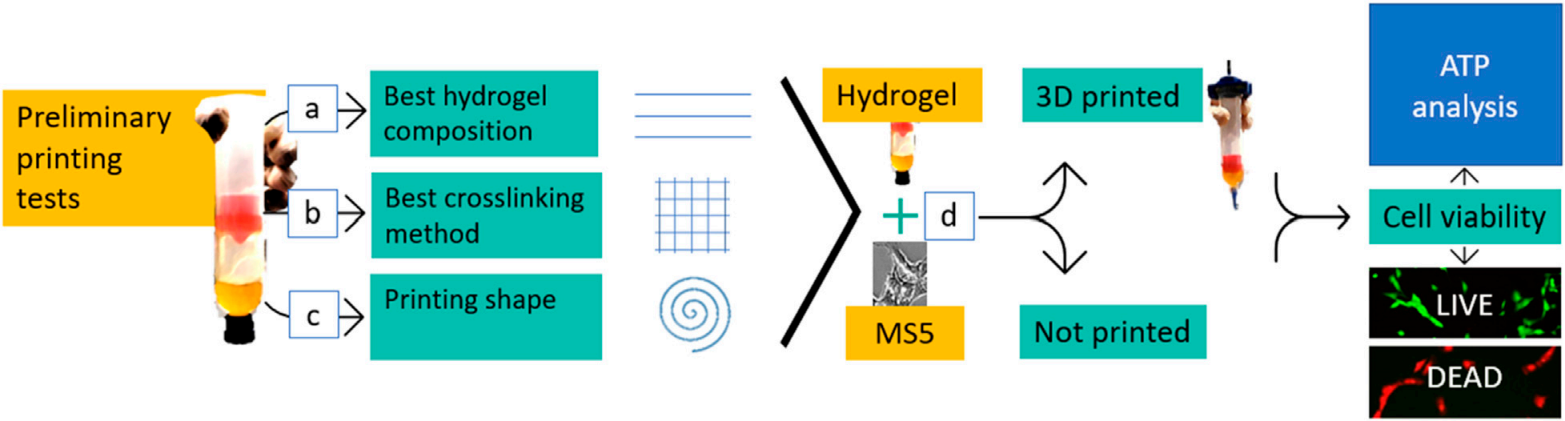
Workflow. Once the hydrogel was produced, three main preliminary printing tests were carried out to define the best ink composition **(A)**, the best crosslinking method **(B)**, and if it was possible to print a shape with the investigated hydrogels **(C)**. The next step consisted in combining the hydrogel with cells (MS5) **(D)**. The mixtures were analyzed plane (not printed) and after the 3D cell printing process. Cell morphologywas assessed, and cell viability was tested by using both the live/dead staining and the ATP assay.

The printing protocol followed for the printability tests was carried out on cell-free hydrogels on three phases: ink composition investigation (Figure 1A and as illustrated in (Gaudenzi et al., 2024), crosslinking method definition (Figure 1B), and 3D structures printing (Figure 1C).

The 3D BIOPLOTTER (Envisiontec ^©^, Gladbeck, Germany) was used for bioprinting. Printing parameters (i.e.,: pressure, velocity and temperature) (Figure 1A), and crosslinking methods (Figure 1B) were varied in the process. A set of experiments of single filament printing was carried out to determine the hydrogel composition. Single filament printing enables a qualitative and quantitative evaluation of the printability, leading to the definition of the optimal parameters. The experiments involved parameters tuning by printing filaments at various pressure, velocity, and temperature values.

Following the filaments printing, different grids consisting of a 30 mm × 30 mm square, with 6 mm of distance between the long parallel lines, were printed to test two crosslinking procedures. The grids replica used for this evaluation were printed with the following parameters: P of 2.3 bar, 0.45 mm offset, nozzle of 0.61 mm, and V of 30 mm/s. In the first scenario, the crosslinking was performed during the printing, in a bath of 3% CaCl_2_ solution, while in the second one the crosslinking was performed after printing for 3 min.

**FIGURE 2:**
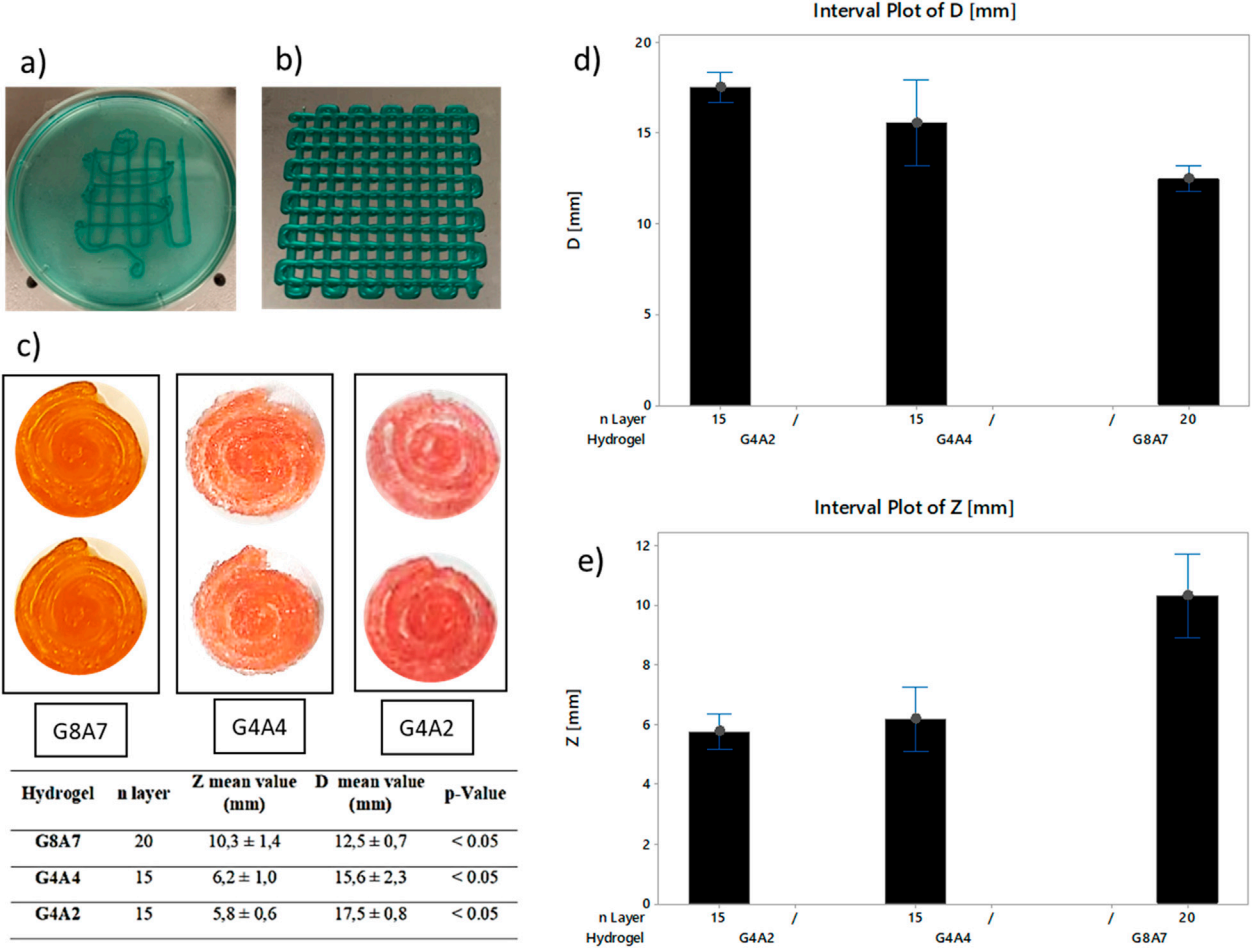
**(A)** Grids printed in bath and **(B)** on a dry surface, pressure 2.3 bar, offset 0.45 mm, and speed 30 mm/s. Pictures have been taken at a magnification of × 1. While printing, the hydrogel was combined with a blue edible dye in order to make the printed geometry visible. **(C)** Top view of the spirals for each hydrogel. G8A7: pressure 1.8 bar, speed 20 mm/s, offset 0.35 mm–20 layers. G4A4: temperature 10°C, speed 20 mm/s, pressure 1 bar–15 layers. G4A2: head temperature 10°C, plate temperature 5°C, speed 20 mm/s, pressure 0.8 bar–15 layers. Summary table: *z* and diameters measurements, and statistical evaluation of the printed structures. **(D)** Diameter measurements for the samples: G8A7 – 20 layers, G4A4 – 15 layers, and G4A2 – 15 layers. **(E)** *z* measurements for the samples: G8A7 – 20 layers, G4A4 – 15 layers, and G4A2 – 15 layers.

Subsequently, a 3D structure (Figure 1C) was printed. The chosen geometry was a spiral, designed as 3.5 revolutions inscribed in a 20 mm diameter circumference (Figure 2C), with 0.5 mm thickness and height of 10 mm. Since the main aim of this experiment was to assure cellular survival after printing, such geometry, with a higher surface ratio, can help with nutrients, oxygen, and other supplements exchange enhancing cell viability and functionality. The structures were printed with 0.35 mm offset and 0.35 mm nozzle and were considered stable when reaching 15 layers without collapsing.

To verify the different percentages of Gelatin and Alginate effects on the measurements of diameter and *z* height of the structures, single-factor analysis of variance (ANOVA) tests were performed. This analysis was used to confirm the statistical significance within samples and to determine repeatability. Statistical analysis was conducted at α = 0.05 using Minitab 18^®^ (MINITAB, Brandon Court Unit E1-E2, Progress Way, Coventry CV3 2TE, United Kingdom).

### 2.3 Viscosity tests

Viscosity analyses were carried out using the VISCOTM 6800 (ATAGO CO., LTD. Tokyo, Japan). Spindle (A1, A2, A3) and beaker (small S) were selected for each experiment, according to the measurable range. All the samples were heated to a temperature of 45°C and then cooled down to 23°C, then poured in the beaker, avoiding air inclusion. Once the hydrogel reached 20°C, the measurement process started. The data were collected using the software Tera Term (Tera Term Project). For each angular velocity, the 10 central measurements were considered for G8A7 and the 20 central measurements for G4A4 and G4A2.

The viscometer registers a viscosity value (mPa*s) at each turn. The standard deviation was calculated for each speed value. Given the beaker inner diameter (DB), the spindle external diameter (DS), and the angular speed in radiant ( ω ), the shear rate is obtained with the below-reported function:

$$Shear\;rate\;\left[s^{-1}\right]=2\frac{D_{B}^{2}}{D_{B}^{2}-D_{s}^{2}}*\omega$$

### 2.4 Cell-laden hydrogels preparation

MS5 murine stromal cells were cultured in DMEM medium (Euroclone, Milan, Italy), supplemented with 10% fetal bovine serum (FBS, Euroclone), 1% penicillin/streptomycin (Euroclone), and 1% L-glutamine (Euroclone), at 37°C, 5% CO_2_ in a humidified incubator, as recommended. MS5 cells were kindly provided by the San Raffaele Telethon Institute for Gene Therapy, Milan, Italy. When reaching confluence, cells were detached using trypsin-EDTA (Euroclone).

For the incorporation in the hydrogel at a density of 4 × 10^5^ cells/mL, cells were counted and centrifuged in a sterile tube (Figure 1D). The pellet was dried and dislodged; then, the hydrogel mixture was gently added to the dry pellet by making it slide down the side of the tube. The cell suspension was homogeneously mixed by gently inverting the tube, and briefly spun down in a centrifuge.

The cell-laden hydrogel was poured in the 3D BIOPLOTTER (Envisiontec ^©^, Gladbeck, Germany) with a sterile cartridge OPTIMUM CLASS VI, 30 cc (Nordson, Westlake, OH); the printing of cell-laden hydrogels was carried out using P below 1 bar (0.8 bar) to preserve cell viability.

To set the not printed hydrogels, part of the cell-laden hydrogel was aliquoted in the wells of a 24-well plate, 500 µL/well, crosslinked with 3% CaCl_2_ for 3 min, and washed twice with DMEM to remove all the traces of CaCl_2_. An equal number of cells was seeded in a 24-well plate in the traditional 2D culture conditions. Both printed and not printed cell-laden hydrogels, as well as 2D culture cells, have been maintained in culture in DMEM medium supplemented as described before, at the same temperature conditions. Cells were observed and pictures were taken with an inverted optical microscope (EVOS™ XL, Thermo Fisher Scientific) to assess cell morphology.

### 2.5 Biological characterization

Cell activity was assessed by two different assays: Live/Dead assay and ATP cell viability luciferase assay (Riva et al., 2022; Mazzoldi et al., 2022; Rovetta et al., 2023; Ferraro et al., 2022).

For the Live/Dead assay, the LIVE/DEAD™ cell imaging kit (488/570) by Thermo Fisher Scientific was used. The kit consists of two components: Calcein AM stains live cells in green, while BOBO-3 iodide stains dead cells in red. The vial with dried BOBO-3 iodide must be resuspended with the vial with calcein AM to obtain a 2 × mix. A small slice of both printed and not printed hydrogels was cut with a sterile scalpel and moved into a centrifuge tube, where it was incubated with complete DMEM and Live/Dead in 1:1 proportion for 15 min at room temperature in the dark. The assay robustness was verified on a control of dead cells (MS5 cells cultured in 2D and fixed in 100% ethanol for 2 min) and a control of live cells (unfixed MS5 cells, cultured in 2D), that appeared red and green, respectively.

The hydrogel slice was then flattened between a glass coverslip and a microscope slide and observed under a fluorescence microscope (Olympus I × 70, Olympus, Tokyo, Japan). Images from at least 5 fields were taken using Image-Pro Plus software v7.0 (Media Cybernetics, Rockville, MD).

For the ATP cell viability luciferase assay, the CellTiter Glo^®^ 3D cell viability assay (Promega, Madison, WI) was used. The assay takes advantage of the ATP produced by living, metabolically active cells to catalyze luciferase-mediated luciferin oxidation, which results in photon emission. After culture medium removal, cell-laden printed and not printed hydrogels (500 µL/specimen) were incubated with 500 µL of the kit substrate for 20 min at room temperature in the dark. During the incubation, the hydrogel was mechanically broken by repeated pipetting and smashing with a syringe plunger to allow cell lysis; the substrate itself contained a lysing reagent optimized for 3D structures. As a positive and a negative control, cells in 2D cultures and the hydrogel without cells were assayed as well. A standard curve was prepared by standard ATP serial dilutions. After the incubation, the mix was moved to a black 96-well plate (BRAND, Wertheim and Großostheim, Germany) for the analysis: for each sample, three replicate wells were set (150 µL/well). After an additional 10 min incubation for bubble removal, the luminescence signal was acquired with a M200 microplate reader (Tecan, Männedorf Switzerland). The analyzed timepoints were: t0 (not printed only), 3 h, 24 h, 72 h, and 7 days. The mean was calculated from the three replicate wells, the signal from the empty hydrogel was subtracted, and the absolute ATP concentration was calculated from the standard curve equation. Data were expressed as percentages of the signal from cells in 2D culture at t0.

GraphPad Prism (San Diego, CA) was used to perform comparisons between groups by *t*-test for data from biological experimental replicates. The number of experimental replicates and the *p*-values are reported in the individual figure legends. All presented data are expressed as mean ± standard deviation, and *p*-values <0.05 were considered statistically significant.

## 3 Results and discussion

### 3.1 Printing, crosslinking and shape optimization

The results demonstrated that the G8A7 showed the best shape fidelity (Gao et al., 2019) in comparison to the other hydrogels. The filaments were well defined and with smooth surfaces while G4A4 and G4A2 produced defined filaments with a not entirely smooth surfaces, but requiring lower pressure values, more suitable for the 3D cell printing applications.

The comparison between the crosslinking during printing (Figure 2A) and post printing (Figure 2B) reveals that, during printing, the grids hydrogel filaments showed poor adhesion, as visible from Figure 2A. This effect is caused by the presence of Alginate that immediately crosslinks with the CaCl_2_, thus the crosslinked filament surface does not stick to the printing substrate. Therefore, crosslinking was performed after printing.

During the experimental tests, the spiral geometry showed geometric stability when printing, despite the continuous filament printing opposed to the traditional start-and-stop geometry of grids with different patterns. In case of grids printing, due to the many stops and starts, the filaments are not continuous, and the resulting layers are uneven, affecting the final geometry. The discontinuities inside the layer are also crucial for cell survival because they can interfere with the nutrients exchange surface, as explained.

Figure 2C shows a representation of the printed spirals; the set parameters were P 1.8 bar and V 20 mm/s at 20°C for G8A7; P 1 bar and V 20 mm/s at 10°C for G4A4; P 0.8 bar and V 20 mm/s at 10°C for G4A2. The measurements of *z* height and diameter showed how the amount of Gelatin affects the stability during the printing phase (Figures 2C–E). G8A7 has higher percentage of Gelatin, so it is stable at higher temperatures. This condition guarantees higher stability of the sample; in particular, the number of layers can be higher, thus the *z*. Moreover, the trend of the diameter is inversely proportional to the Gelatin content. G4A2 and G4A4 hydrogels are less stable during the printing phase, so the diameter of the printed structures tends to expand due to the load of the upper printed layers.

The results indicate that the G8A7 hydrogel can be printed more successfully in terms of structural integrity, but in order to be used in 3D cell printing, the hydrogel needs to have certain rheological and biological properties. The composition of cell-laden hydrogels significantly affects cell viability and proliferation, even when using materials deemed biocompatible. Recent research highlights that factors such as the material content and stress from the bioprinting process play crucial roles in cell survival. For instance, hydrogel materials with higher polymer content or stiffness can exert mechanical stress on encapsulated cells, potentially impairing their function (Žigon-Branc et al., 2019). Additionally, the bioprinting process itself introduces shear forces that can impact cell viability. Hence, the hydrogel formulation has to be adjusted as well as the experimental conditions to maintain cell health and function (Luo et al., 2022; Yue et al., 2024).

### 3.2 Viscosity

**FIGURE 3:**
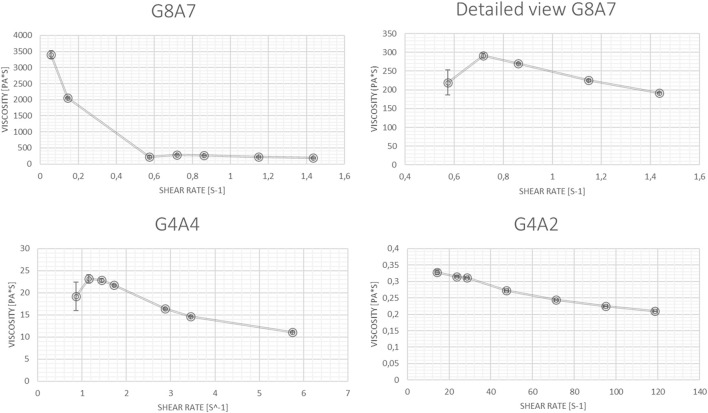
Viscosity as shear rate’s function. Resulting trends for G8A7, G4A4, G4A2.

The main results about viscosity as function of the shear rate are collected in Figure 3.

The viscosity magnitude order is:

− 10^3^ Pa*s for G8A7 when the shear rate is lower than 0.57 s^−1^ and 10^2^ for bigger shear rate values;
− 10^1^ Pa*s for G4A4 for shear rate values between 0.85 and 8.62 s^−1^;
− 10^−1^ Pa*s for G4A2 for shear rate values between 14.24 and 118.69 s^−1^.

Indeed, for common shear rate values (in the range between 0.85 and 1.73 s^−1^), a difference of one order of magnitude in viscosity is noticed for G8A7 and G4A4. The viscosity values recorded for G4A4 are comparable to the ones found in literature for similar hydrogels (Liu et al., 2019; Mondal et al., 2019; Fayyazbakhsh et al., 2022; Ninan et al., 2014). The viscosity order of magnitude reported in (Fayyazbakhsh et al., 2022) for the 4% (w/v) Alginate and 4% (w/v) Gelatin (type B) hydrogel is the same recorded by the authors for G4A4 while the differences observed in the values can be attributed to the use of different material types (Gelatin type A vs. type B (Fayyazbakhsh et al., 2022)). Viscosity values around zero were registered in (Mondal et al., 2019) at shear rate values bigger than 20 s^−1^ for the hydrogel with the following composition: 3% (w/v) Gelatin (type B) and 4% (w/v) sodium Alginate (50,000 Da), mixed within a 0.45% saline solution. So, the 10^−1^ order of magnitude obtained in this work for G4A2 can be considered compatible with this composition.

Finally, the maximum viscosity obtained in (Liu et al., 2019) for a 15% (w/v) Gelatin and 8% (w/v) Alginate (80,000–120000 g/mol vs. 120,000–190000 g/mol used by the authors in this work) is near 10^2^ Pa*s. Hence, according to (Liu et al., 2019) and observing that, in accordance with (Gong et al., 2020) and (Maihemuti et al., 2023), Alginate can be considered more significant than Gelatin for determining viscosity, (Maihemuti et al., 2023), the results here reported for G8A7 (orders of 10^2^ and 10^3^ Pa*s depending on the shear rate) are consistent. Hydrogel viscosity has a profound impact on cell viability and metabolic activity. Recent findings indicate that hydrogels with high viscosity (>10^2^ Pa*s) can form stable 3D structures, but they may adversely affect cell metabolic activity (Venkata Krishna and Ravi Sankar, 2023; Sughi et al., 2023). For instance, despite cells appearing alive in live/dead assays, ATP analysis often reveals significantly reduced metabolic activity compared to 2D controls (Masson-Meyers et al., 2016). This disparity highlights that while high-viscosity hydrogels provide structural integrity, they may hinder cellular function due to limited nutrient and oxygen diffusion. Therefore, optimizing hydrogel viscosity is crucial for balancing structural stability and cellular functionality.

### 3.3 Biological results

G8A7, G4A4, and G4A2 cell-laden hydrogels have been tested for their biocompatibility both as printed and not printed hydrogels.

**FIGURE 4:**
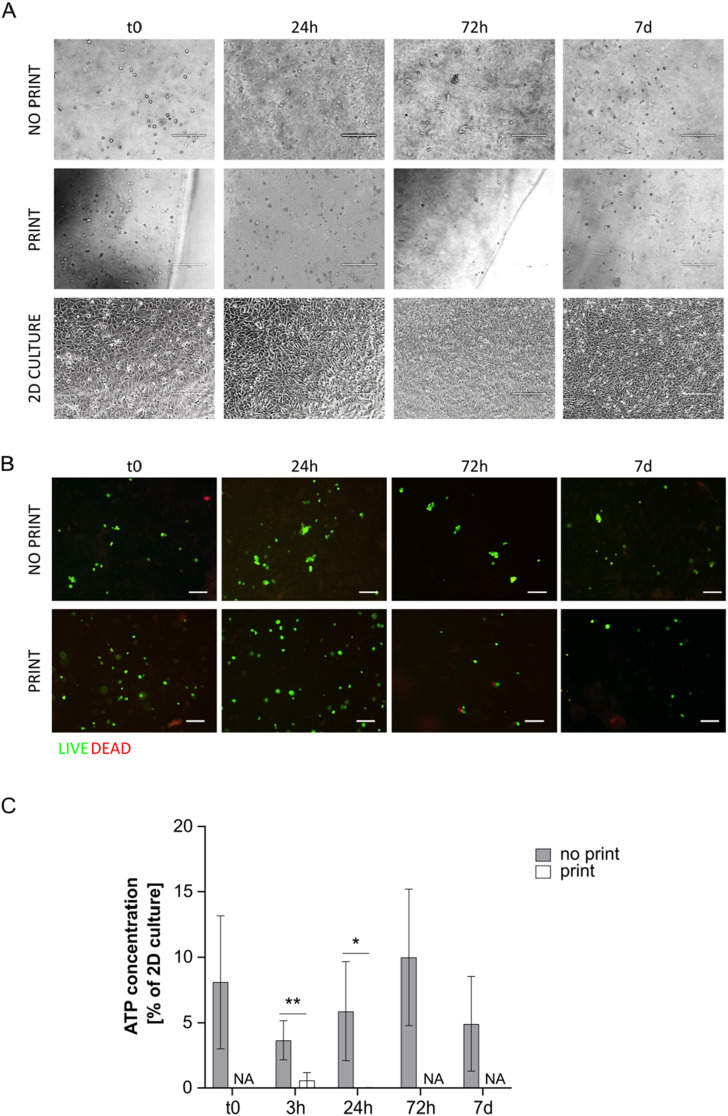
**(A)** Representative pictures of MS5 cells in not printed and printed G8A7 and in 2D culture at day 0, 24 h, 72 h, and 7 days. Scale bar 200 µm. **(B)** Live/Dead assay on cell-laden G8A7 hydrogel (both not printed and printed) at t0, 24 h, 72 h, and 7 days. Representative pictures are shown. Scale bar: 100 μm. **(C)** ATP concentration measurement, expressed as percentage of MS5 cells in 2D culture at t0, in cell-laden G8A7 hydrogel (both not printed and printed) at t0, 3 h, 24 h, 72 h, and 7 days. Results are expressed as mean ± standard deviation (N = 3 print; N = 4 no print); *p < 0.05, **p < 0.01 print vs. no print, Student’s t-test. NA = not available.

Cells were clearly visible in the G8A7 hydrogels, both printed and not printed, under microscope inspection; however, they appeared round-shaped also many days after hydrogel incorporation, meaning that cells were not able to spread and to assume their usual morphology, probably because they could not move inside the medium due to its excessive stiffness and low degradation rates (Figure 4A).

Printed and not printed cell-laden G8A7 hydrogels were subjected to live/dead staining. Both presented a vast majority of green spots at all the analyzed timepoints. The red spots were very rare or even undetectable, meaning that almost all the cells were alive (Figure 4B), which does not directly exclude metabolic quiescence or cell senescence.

On the contrary, ATP test revealed that metabolic activity was very low: indeed, ATP content was always under 15% of 2D culture control in not printed hydrogels. The trend started with a strong reduction at 3 h, probably as a consequence of adaptation, followed by a slight increase at 24 h and 72 h; again, the signal was strongly reduced at day 7. Nonetheless, the observed differences among timepoints are not statistically significant, due to high variability among replicates. The situation was critical for the printed hydrogels: at 3 h after incorporation (immediately after printing), the metabolic activity was around 0.6% of 2D culture control, and it dropped down to overlap the empty hydrogel at 24 h. To this reason, it was not worth going on with later timepoints (Figure 4C).

The poor metabolic activity may be a consequence of the fact that the formulation resulted highly viscous and difficult to handle, since it was fluid within a small temperature window. In addition, the reproducibility of the results was negatively influenced by the difficulty to manage precise volumes, a necessary step to perform the assay.

**FIGURE 5:**
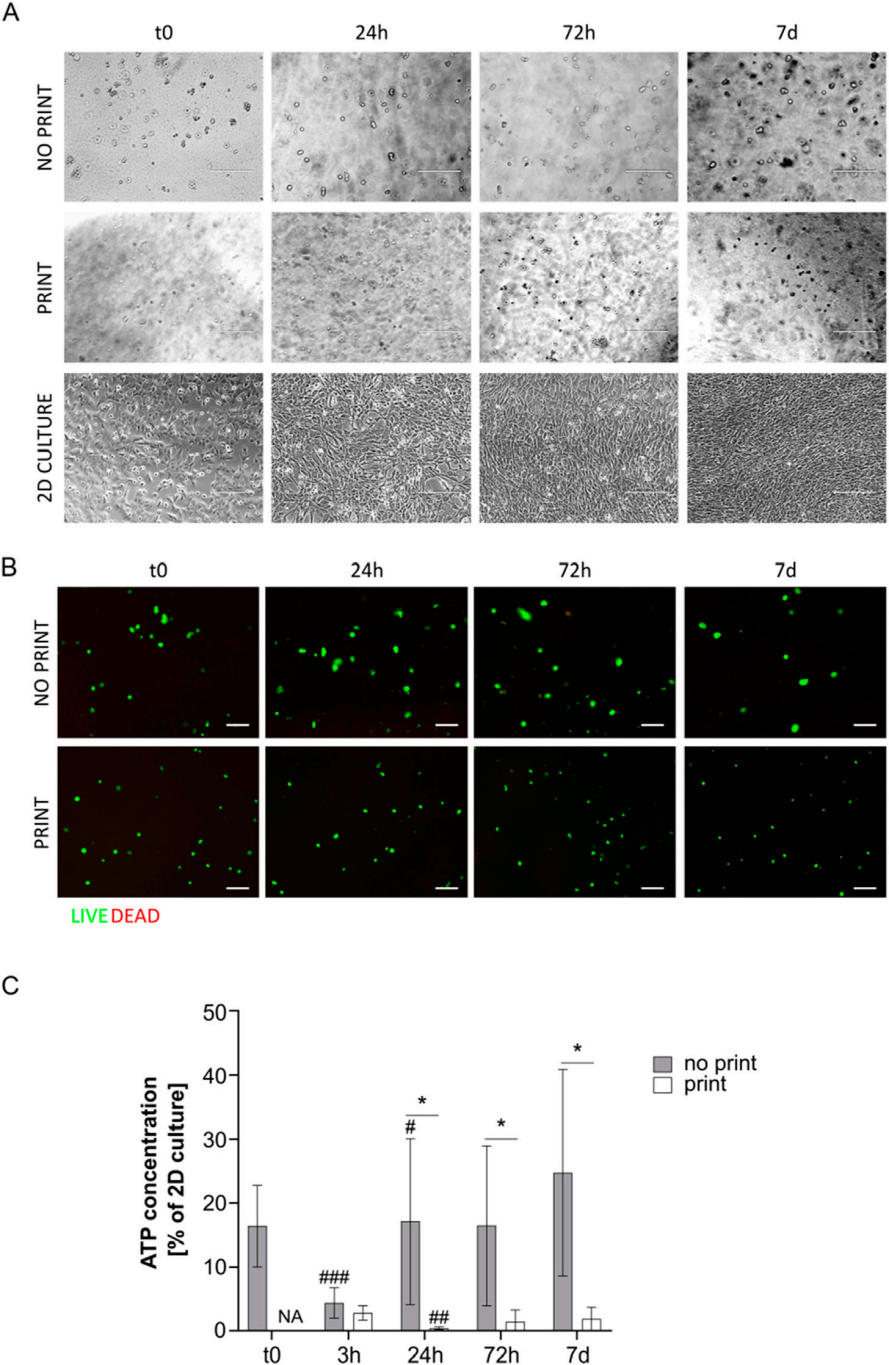
**(A)** Representative pictures of MS5 cells in not printed and printed G4A4 Gelatin and in 2D culture at day 0, 24 h, 72 h, and 7 days. Scale bar 200 µm. **(B)** Live/Dead assay on cell-laden G4A4 hydrogel (both not printed and printed) at t0, 24 h, 72 h, and 7 days. Representative pictures are shown. Scale bar: 100 μm. **(C)** ATP concentration measurement, expressed as percentage of MS5 cells in 2D culture a t0, in cell-laden hydrogel G4A4 (both not printed and printed) at t0, 3 h, 24 h, 72 h, and 7 days. Results are expressed as mean ± standard deviation (N = 7 no print, N = 4 print); *p < 0.05 print vs. no print; #*p* < 0.05, ##*p* < 0.01, ###*p* < 0.001 vs. previous timepoint, Student’s t-test. NA = not available.

Similarly to G8A7 hydrogel, the G4A4 hydrogel did not allow cell spreading. Indeed, cells appeared round-shaped at microscopic inspection with this formulation (Figure 5A).

Live/dead staining revealed again that almost all the cells were green (*i.e.*, alive) in both printed and not printed hydrogels (Figure 5B). An interesting observation is that the green spots appeared bigger in the not printed hydrogel, suggesting that cells presented a wider cytoplasm and thus were considered in a healthier state. However, it must be stated that the cell density observed in the representative pictures here presented is not indicative of the actual cell number in 3D culture, since the protocol developed requires sample cutting, manipulation, and flattening to allow the staining and the acquisition.

Nonetheless, ATP test revealed low metabolic activity in the not printed hydrogel and even lower in the printed one. However, the not printed hydrogel outcome was better in comparison to the G8A7 hydrogel: indeed, the ATP content at t0 was around 16% of MS5 cells in 2D culture conditions. The trend was similar to the previously tested hydrogel: at 3 h, the ATP signal dropped down; the ATP content increased again starting from 24 h, reaching 17%, then it remained quite stable for the subsequent timepoints, reaching a peak at day 7, around 25% of 2D culture. The differences between t0 and 3 h and between 3 h and 24 h are statistically significant, while no significance was recorded between 24 h and 72 h and between 72 h and 7 days.

In the printed hydrogel, 3 h after incorporation (immediately after printing), the metabolic activity was comparable to the not printed one. We can exclude it is a consequence of thermal shock (bioprinting was here performed at 10°C) since preliminary tests highlighted a higher percentage of living cells when MS5 cells were kept at 10°C than at 37°C in non-adherent conditions (data not shown) and because cells in 2D culture control and in the not printed hydrogel were kept at 10°C as well. However, the ATP content strongly decreased at 24 h, since the signal was comparable to the empty hydrogel. Nonetheless, it started increasing again, but it settled on 1.4%–1.9% at 72 h-7 days, meaning that cells failed the adaptation process (Figure 5C).

The results related to the not printed hydrogel may be accounted to the fact that the G4A4 hydrogel was less viscous (as demonstrated) and easier to handle, and it could stay fluid within a broader temperature window. Nonetheless, the shear stress related to the printing process was enough to prevent cells to survive and proliferate.

**FIGURE 6:**
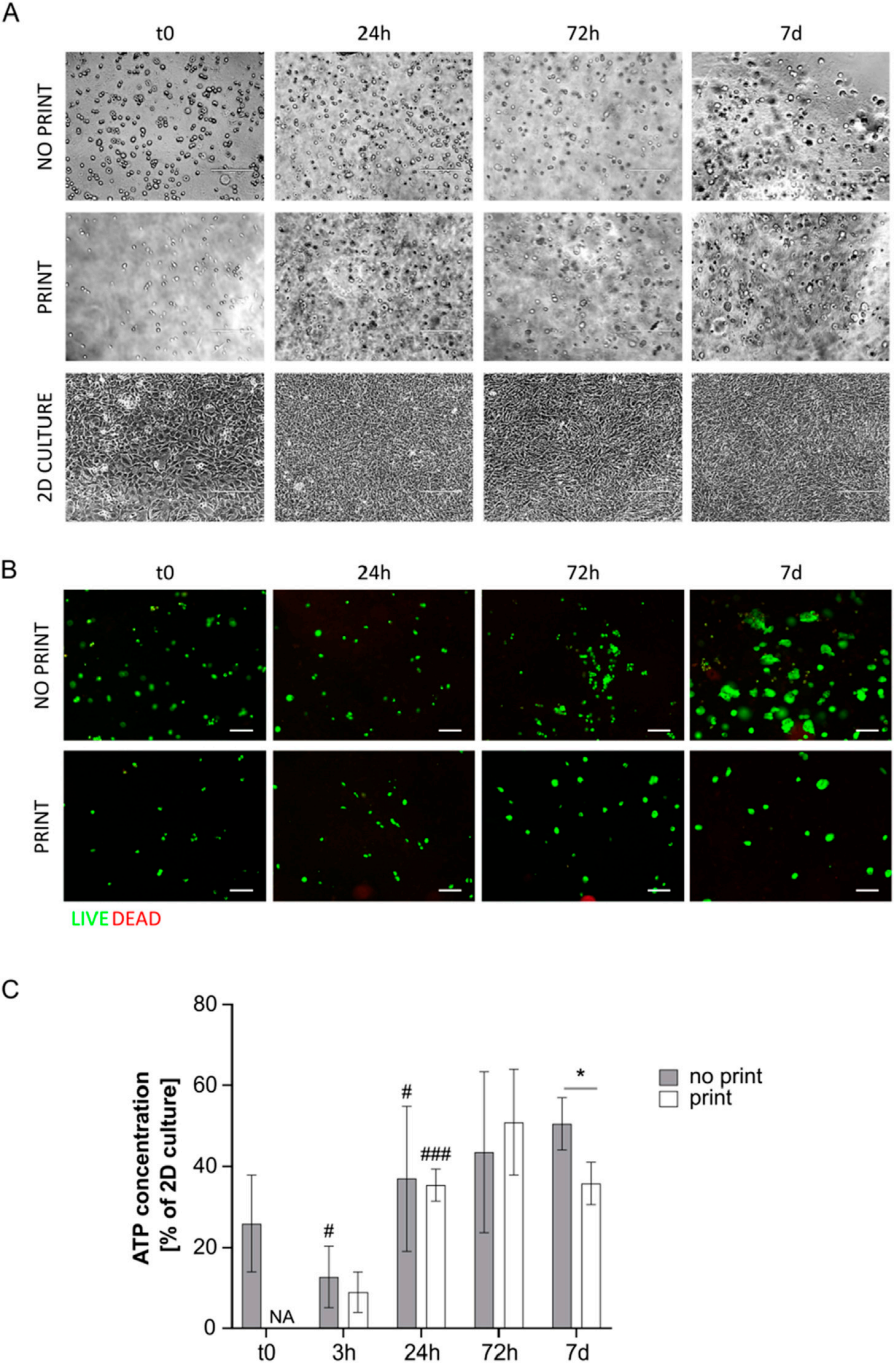
**(A)** Representative pictures of MS5 cells in not printed and printed G4A2 and in 2D culture at day 0, 24 h, 72 h, and 7 days. Scale bar 200 µm. **(B)** Live/Dead assay on cell-laden G4A2 hydrogel (both not printed and printed) at t0, 24 h, 72 h, and 7 days. Representative pictures are shown. Scale bar: 100 μm. **(C)** ATP concentration measurement, expressed as percentage of MS5 cells in 2D culture a t0, in cell-laden G4A2 hydrogel (both not printed and printed) at t0, 3 h, 24 h, 72 h, and 7 days. Results are expressed as mean ± standard deviation (N = 4 no-print, n = 3 print); *p < 0.05 print vs. no print; #*p* < 0.05, ###*p* < 0.001 vs. previous timepoint, Student’s t-test. NA = not available.

Even if the G4A2 hydrogel resulted the less viscous, similarly to the previously tested formulations, did not allow cell spreading. Indeed, cells appeared round-shaped at microscopic inspection (Figure 6A).

Live/dead staining revealed again that almost all the cells were alive. Moreover, the green spots appeared bigger, with a wider cytoplasm, especially in the not printed specimens and at later timepoints (Figure 6B).

The ATP test revealed that the present formulation was the promising among the tested ones. However, the metabolic activity barely reached 50% of the activity of MS5 in 2D culture conditions in plastic vessels. Indeed, at t0, the not printed hydrogel showed an ATP content around 25% of the 2D culture. It dropped at 3 h and increase again at 24 h, reaching 37%. Between 24 h and 7 days, the ATP content was stable, around 40%–50% of 2D culture. Regarding the printed hydrogel, the ATP content was about 10% of 2D culture immediately after printing, displaying no significant difference with the not printed one, but it started increasing at 24 h (35%). It increased again at 72 h, yet not significantly, while it started slightly decreasing at day 7. It is interesting to notice that at 24 h and at 72 h no significant difference between printed and not printed samples was observed (Figure 6C).

Since the percentage of Alginate was brought to 2%, while the percentage of Gelatin was maintained constant at 4%, the better results in terms of cell survival and proliferation may be related to the reduced shear stress on cells while printing, due to the reduced viscosity of the medium (10^−1^ Pa*s vs. 10^1^ Pa*s and 10^2^–10^3^ Pa*s for G4A4 and G8A7, respectively). Indeed, it has been previously reported that Alginate determines the major effect on viscosity (Gong et al., 2020; Maihemuti et al., 2023). Moreover, Alginate is the main responsible for cell toxicity in bioinks, since it is the substrate of the crosslinking agent, *i.e.*, CaCl_2_. The results replicability also increased: the G4A2 could be easily managed and then disaggregated to perform ATP test. The pipetted volumes were much more precise in comparison to the previous G8A7 and G4A4 hydrogels.

In Table 1, a direct comparison between cell viability at day 7 and the hydrogels viscosity order of magnitude is presented.

**TABLE 1:** Direct comparison between hydrogels’ cell viability at 7 days and viscosity order of magnitude. Cell viability is reported as % of metabolic activity, compared to the 2D culture. Viscosity is reported in Pa*s.

Hydrogel (A)	Cell viability at 7d (%) printed	Cell viability at 7d (%) not printed	Viscosity’s order of magnitude (Pa*s)
G87	N.A.	4.91	10^2^–10^3^
G44	1.87	24.74	10^1^
G42	35.76	50.57	10^−1^

A clear relation between viscosity and cell viability is observed. This relation can be attributed both to a higher shear stress acting on the cells in the syringe due to a higher viscosity of the hydrogels, and to the conditions where the cells have to survive, move and interact. As shown, low viscosity values allow higher cell viability, probably due to a better exchange of nutrients and higher cell motility which enhance the cells communication and proliferation. The bioprinting process introduces various stresses that can impact cell viability and activity. Studies show that bioprinting can initially reduce cell viability and metabolic activity by up to 20% compared to non-printed hydrogels (Ning et al., 2018). This reduction is attributed to shear stress during printing and the time required for cells to adapt to the new environment. Despite this initial drop, printed hydrogels can achieve cell activity levels up to 50% of those in traditional 2D cultures once the cells have adapted (Ouyang et al., 2015). This adaptation period and the associated recovery highlight the need for careful optimization of printing parameters to minimize stress and support cell survival (Theus et al., 2020).

## 4 Conclusion

The aim of this work was to evaluate the cell activity in relation to printed and not printed easy-to-make hydrogel that mimics a connective tissue, such as vitreous body, dermis, or adipose tissue, in terms of cells survival, spreading, and metabolism. However, the production of hydrogels that guarantee structural stability and cell survival at the same time is complex. Indeed, the production processes of such bioinks are hardly repeatable and cell viability depends on different factors (crosslinking, shear stress during extrusion, hydrogel viscosity, printing parameters: pressure, velocity, head temperature, plate temperature, presence of the so-called integrin-binding sites). To reach this goal, it was necessary to first optimize the printing conditions and then to test cell activity for the proposed hydrogels.

The results of this work that can be considered a step forward in the state of the art have been highlighted:

– The cell viability cannot be measured from imaging analysis but should be reported as a combination of viable cell percentage, cell metabolic activity and cell morphology. Live/Dead staining and ATP analysis do not provide the same results in terms of cell activity: indeed, the two assays provide complementary information regarding cell survival, metabolic activity, and proliferation.
– The composition of cell-laden hydrogels can affect cells viability and proliferation even if considered biocompatible. Major factors are the materials content (percentage) and the stress related to the bioprinting process.
– The bioprinting process of cell-laden hydrogels is a valid alternative for producing tissue engineered models with specific requirements, not in substitution for traditional 2D cultures.
– Viscous hydrogels (>10^2^ Pa*s) can produce a stable 3D structure, but the cells seemed alive using the live/dead kit, while the ATP analysis showed poor metabolic activity (5% of 2D control).
– Differences in cell viability are evident moving from a hydrogel viscosity of 10^−1^ to a viscosity of 10^2^ Pa*s. Low viscosity values (<10^1^ Pa*s) result in higher cell activity (50% of 2D control).
– The printing process initially lowers cell viability and activity up to the 20% in relation to not printed hydrogels, that already express a cell activity that reaches the 50% of the 2D culture as maximum value; this is probably as a consequence of shear stress and of longer adaptation time.

From these results it can be concluded that the hydrogels biocompatibility characterizations reported in the related literature should be critically evaluated, since the most used assay to assess cell viability is the Live/Dead staining for imaging, often limited to early timepoints (t0, 24 h). In addition, cells are usually 3D-printed at a density of several millions of cells/mL (Li et al., 2018; Gregory et al., 2022). This strategy could be good for organs like the heart, kidneys, liver, but not for connective tissues with cell densities ranging from 1 × 10^5^ to 8 × 10^5^ cells/mL.

Printing of extremely high cell densities may result in a good cell survival; however, the actual viable cells percentage of the structure should be considered in relation to the initial amount of cells, not to force any phenomena by overpopulating the bioink. In fact, the cells initial number should be selected considering the tissue that is meant to be reproduced, in order to validate each 3D printed cell-laden model. Further research will investigate other hydrogel compositions, to further evaluate the viscosity effects and the metabolic activity trend in relation to hydrogel compositions that are easy-to-make and require the lowest amount of components as possible, also to prevent side effects. A possible solution to use the present composition at even lower concentration and viscosity to further improve cell viability could be the developing of other crosslinking baths to support the 3D structure stability, *i.e.*, the Freeform Reversible Embedding of Suspended Hydrogels (FRESH) method (Bessler et al., 2019; Kreimendahl et al., 2021; Seiti et al., 2023), that exploits processed Gelatin as a support bath that can crosslink the hydrogel during printing. In the present work, a generic commercial stromal cell line, easy to culture and low-cost, was employed as a proof-of-concept, similarly to what is done in literature with NIH-3T3 murine fibroblasts. Future research will lead to develop more specific *in vitro* 3D tissue models, which will exploit the use of tissue specific cell types, such as cells obtained from pluripotent stem cell differentiation.

## Data Availability

The raw data supporting the conclusions of this article will be made available by the authors, without undue reservation.
